# Flexible Nonvolatile Bioresistive Random Access Memory with an Adjustable Memory Mode Capable of Realizing Logic Functions

**DOI:** 10.3390/nano11081973

**Published:** 2021-07-31

**Authors:** Lu Wang, Yukai Zhang, Dianzhong Wen

**Affiliations:** HLJ Province Key Laboratory of Senior-Education for Electronic Engineering, School of Electronic Engineering, Heilongjiang University, Harbin 150080, China; 2191278@s.hlju.edu.cn (Y.Z.); wendianzhong@hlju.edu.cn (D.W.)

**Keywords:** tussah hemolymph, bioresistive random access memory, bipolar resistive switching, WORM, logic gate, flexible

## Abstract

In this study, a flexible bioresistive memory with an aluminum/tussah hemolymph/indium tin oxide/polyethylene terephthalate structure is fabricated by using a natural biological material, tussah hemolymph (TH), as the active layer. When different compliance currents (Icc) are applied to the device, it exhibits different resistance characteristics. When 1 mA is applied in the positive voltage range and 100 mA is applied in the negative voltage range, the device exhibits bipolar resistive switching behavior. Additionally, when 1 mA is applied in both the positive- and negative-voltage ranges, the device exhibits write-once-read-many-times (WORM) characteristics. The device has good endurance, with a retention time exceeding 10^4^ s. After 10^4^ bending cycles, the electrical characteristics remain constant. This memory device can be applied for “AND” and “OR” logic operations in programmable logic circuits. The prepared flexible and transparent biomemristor made of pure natural TH provides a promising new approach for realizing environmentally friendly and biocompatible flexible memory, nerve synapses, and wearable electronic devices.

## 1. Introduction

In recent decades, storage limitations have been considered one of the main issues affecting the computing speed of silicon-based electronic devices [1]. The current challenges faced by traditional computer architectures include storage bottlenecks and the high transmission cost between memory and processors, which is called the von Neumann bottleneck. To overcome this bottleneck and increase the calculation speed of such systems, new electronic equipment needs to be developed. For this purpose, resistive random access memory (RRAM) has several potential advantages over traditional electronic devices, such as higher storage density, better stability, and low power consumption [2,3]. The distinctive characteristic of RRAM is that it has different resistance states under different external voltages, enabling its use in many applications, including storage, neural networks, and general memory for computing. Most importantly, an RRAM device can realize the functions of storage and logical calculation simultaneously. RRAM is considered a potential candidate solution for overcoming the von Neumann bottleneck [4]. Emerging 2D materials have received more and more attention in the preparation of memristors due to their unique structures and properties, such as flexible and wearable devices, and neural networks [5,6,7]. Memory devices prepared with metal oxides [8,9,10,11], organic polymers [12,13,14,15], perovskites [16,17,18,19], and other materials as active layers still have major deficiencies in terms of sustainable use and biocompatibility. Therefore, RRAM devices, with their storage and logical computing functions, biocompatibility, and environmental friendliness, are needed to break the von Neumann bottleneck.

After millions of years of evolution, natural biomaterials have developed almost perfect structures and functions, among which electron transfer is one of the most basic processes [20]. Biomaterials have the characteristics of being degradable, transparent [21,22,23,24], and compatible with many substrates. Thus, biomaterials have been increasingly studied by researchers for the preparation of electronic skins [25], organic transistors [26], artificial synapses and neurons [27,28,29], etc. In particular, biomaterials based on proteins [30,31,32,33], DNA [34,35,36], RNA [37], carbohydrates [38], etc., have been used as active layer materials for RRAM. RRAM based on an egg albumen film has been shown to have obvious bipolar resistive switching characteristics [39]. Memory devices using silk fibroin as the active layer exhibit bipolar switching characteristics with an ON/OFF current ratio exceeding 10^4^ [40]. Memory devices based on aquatic lotus plant leaves exhibit nonvolatile resistive switching behavior [41]. Memory devices based on a mixture of starch and chitosan can be used to simulate nerve synapses during the set/reset process [42].

Compared with traditional equipment, the manufacturing process for flexible memory is simpler and lower in cost. Therefore, it is necessary to carry out related research work in this field. In this regard, polyethylene terephthalate (PET) has many excellent characteristics, including a smooth surface and good abrasion resistance. Therefore, PET has become a popular substrate material. A memory device made of pectin on a PET substrate exhibits multilevel storage characteristics [43]. Resistive switching memory devices based on silk protein and Au nanoparticles prepared on PET substrates exhibit bistable electrical switching behavior [44]. Tussah hemolymph (TH) is a natural biological material that can be obtained from tussah organisms. TH is easy to obtain, does not pollute the environment, and is well tolerated by the human body. In tussah larva, oxygen, carbohydrates, protein, and other substances necessary for survival are transported to the organs of the whole body through hemolymph. TH contains substances such as SP-1 and SP-2, which are considered to be a storage reservoir of nutrients and energy [45]. Thus, TH achieves the necessary prerequisites for the application of RRAM made from TH in the human body. In this study, flexible resistive memory devices with an Al/TH/ indium tin oxide (ITO)/PET structure were fabricated by using TH as the active layer and flexible PET as the substrate. The influence of the compliance current on the electrical characteristics of the devices and the influence of bending were investigated. Logical functionality was realized by using the write-once-read-many-times (WORM) device characteristics.

## 2. Materials and Methods

### 2.1. Device Fabrication

The TH used in this study was directly extracted by cutting tussah larvae’s feet. PET substrates coated with ITO electrodes were successively placed in acetone, ethanol, and deionized water for 20 min each for ultrasonic cleaning to remove oil stains and other impurities attached to the surface. TH was then spin coated onto the ITO at a speed of 500 rpm for 5 s and then at a higher speed of 3000 rpm for 40 s, and the formed TH film was dried at 80 °C for 10 min. An aluminum electrode was deposited on the active layer of each device via the thermal evaporation method (under a vacuum of 2 × 10^−3^ Pa) by means of a shadow mask. Each circular aluminum electrode had a diameter of 1.5 mm and a thickness of about 200 nm. Finally, the devices were annealed at 105 °C for 10 min to complete the preparation of flexible bioresistive random access memory with an Al/TH/ITO/PET structure.

### 2.2. Characterization

The electrical properties of the fabricated TH-based RRAM were tested using a semiconductor parameter tester (Keithley 4200, Keithley, Solon, OH, USA). During the electrical testing of each device, the ITO bottom electrode was grounded, while an electrical bias was applied to the aluminum top electrode. The surface morphology of the TH was observed via transmission electron microscopy (JEM-2100, JOEL, Tokyo, Japan). The ultraviolet-visible (UV-Vis) spectrum of TH was obtained with a UV-Vis spectrophotometer (TU-1901, Puxi, Beijing, China).

## 3. Results and Discussion

**Figure 1:**
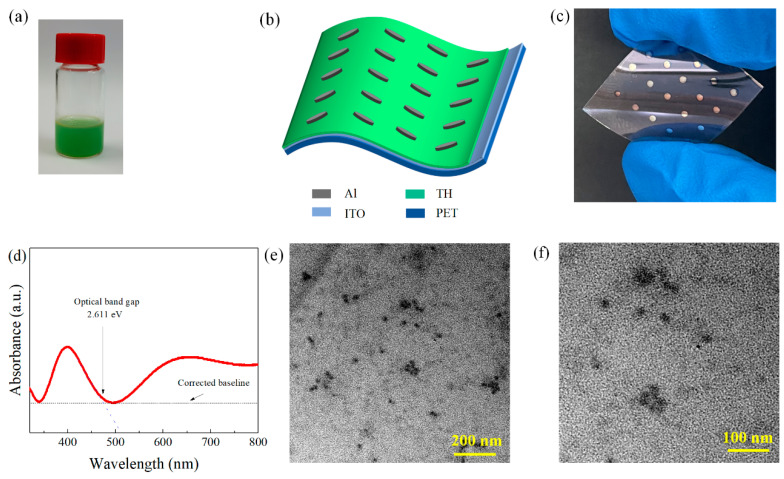
(**a**) TH directly obtained from silkworm larvae. (**b**) structure of the Al/TH/ITO/PET device. (**c**) photograph of the Al/TH/ITO/PET device. (**d**) UV-Vis absorption spectrum of TH. TEM images of TH magnified (**e**) 20,000 times and (**f**) 40,000 times.

Figure 1a shows a photograph of TH. The TH-based flexible RRAM devices were fabricated by means of spin coating and vacuum evaporation technology. An Al electrode was used as the top electrode of each device, and an ITO electrode was used as the bottom electrode. A schematic diagram of the device structure is shown in Figure 1b. A photograph of a fabricated device is shown in Figure 1c, showing its transparency and flexibility. The UV spectrum of the TH material is shown in Figure 1d, from which λ=475 nm can be obtained. Through the formula $E_{g}=\mathrm{hc}/\lambda$ (where h is Planck’s constant, λ is the wavelength, and c is the speed of light), the optical band gap of the material can be calculated to be 2.611 eV. Transmission electron microscopy (TEM) images of the TH material were also obtained, as shown in Figure 1e,f, which presents images magnified 20,000 and 40,000 times.

**Figure 2:**
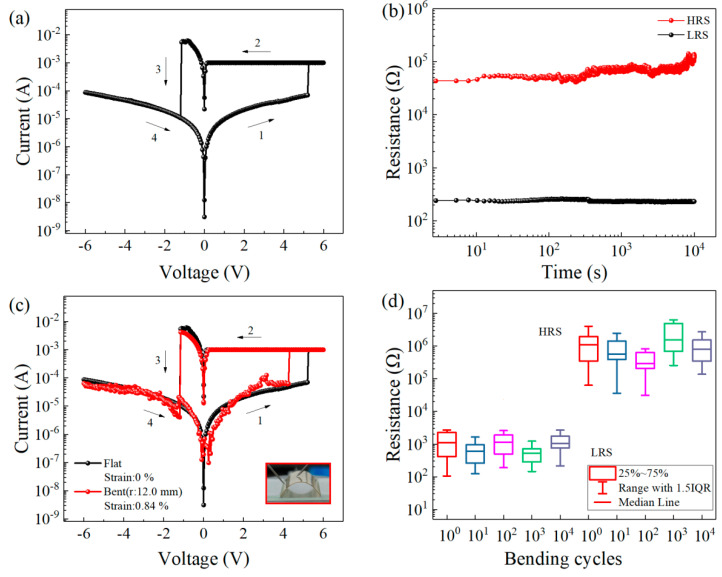
Al/TH/ITO/PET device: (**a**) current-voltage characteristic curve at the applied compliance current in the positive scan area. (**b**) retention time of the device. (**c**) I–V characteristic curve (flat and curved) when the compliance current is applied in the positive voltage region. (**d**) relationship between the number of bending cycles and the resistance state of the device.

To avoid damaging the devices by applying an excessive current, during device testing, a compliance current of 1 mA was applied in the positive voltage scan range, and a compliance current of 100 mA was applied in the negative voltage scan range. The typical current-voltage characteristic curve of an Al/TH/ITO/PET device is shown in Figure 2a. During testing, the bottom electrode of the device remained grounded, while the voltage sweep range was set to −6 V to 6 V. The voltage scan was applied in the direction of the arrows shown in Figure 2a. The device exhibited bipolar resistive switching behavior. The initial state of the device was the high-resistance state (HRS). First, a voltage scan of 0 V to 6 V was applied to the top Al electrode. When the voltage reached the set voltage (V_SET_ = 5.20 V), the device switched from the HRS to the low-resistance state (LRS). This process is called the SET operation. Subsequently, when a voltage scan from 6 V to 0 V was applied, the device remained in the LRS. Next, a voltage scan from 0 V to −6 V was applied. When the voltage reached the reset voltage (V_RESET_) = −1.15 V, the device switched from the LRS to the HRS. This process is called the RESET operation. Finally, when the voltage was subsequently scanned from −6 V to 0 V, the device remained in the HRS.

We tested the retention time of the device at a constant voltage of −0.50 V, as shown in Figure 2b. The results show that the device maintained a good HRS and LRS during the 10^4^ s testing process. Furthermore, no significant attenuation was observed, indicating that the device exhibited the capability of long-term data retention.

Flexible electronic devices play an important role in the manufacture of implantable electronic devices and wearable devices. To test the applicability of the proposed device as flexible memory, the I-V characteristic curves were recorded when the device was flat and bent, as shown in Figure 2c. The strain applied to the substrate was 0.84%, which was determined using the following equation [46].

Strain (%) = (Total thickness of device)/(2 × radius of curvature) × 100(1)

where the total thickness of the device is 200.05 µm which consists of four parts: Al electrode (200 nm), TH film (100 nm), ITO electrode (200 nm), and substrate (200 μm). The strain of 0.84% which is applied to the device is the maximum withstand value of the device, and the strain corresponds to radius of curvature of 12.0 mm. When the device was bent, the resistive switching behavior was consistent with that when the device was flat. To test the bending resistance of the devices, they were subjected to 10^4^ cycles of bending, after which all cells of each device still exhibited bipolar resistive switching behavior. Figure 2d shows the relationship between the number of times a device was bent and the HRS and LRS of the device. The HRS and LRS remained clear and distinct after multiple bending cycles. These experimental results show that the proposed device has excellent mechanical stability and is suitable for flexible storage applications.

**Figure 3:**
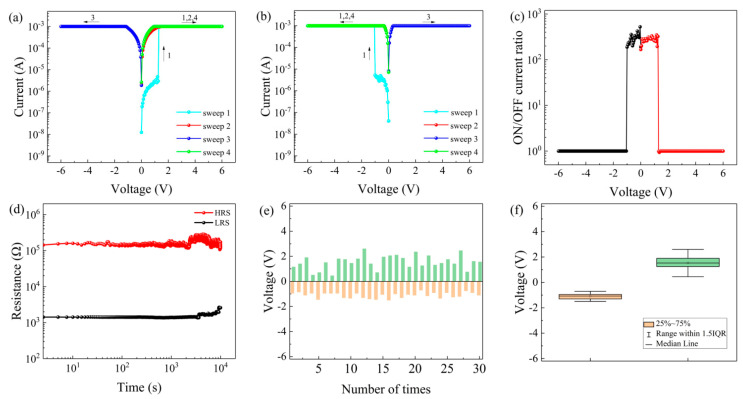
Current-voltage characteristics of the Al/TH/ITO/PET device: (**a**) initially applied negative voltage, (**b**) initially applied positive voltage, (**c**) ON/OFF current ratio of the device, (**d**) retention time of the device, (**e**) threshold voltage histogram and (**f**) threshold voltage statistics graph.

The I-V characteristic curve of a device recorded when a compliance current of 1 mA was applied in both the positive and negative voltage regions is shown in Figure 3a. During the first positive voltage sweep from 0 V to 6 V (sweep 1), the device was initially in the HRS. As the applied voltage increased, the current suddenly switched from 2.95 × 10^−6^ A to 1.00 × 10^−3^ A at V_SET_ = 1.25 V. Consequently, the device changed from the HRS to the LRS, corresponding to the writing process. Under subsequent scans of 0→6 V, 0→−6 V, and 0→6 V, the device remained in the LRS, demonstrating that the Al/TH/ITO/PET device exhibited WORM characteristics. Similarly, as shown in Figure 3b, during the first negative voltage sweep from 0 V to −6 V (sweep 1), when the externally applied voltage reached V_SET_ = −1 V, the device switched from the HRS to the LRS. Then, once the resistance state had switched to the LRS, it remained unchanged during the next three scans (0→−6 V, 0→6 V, and 0→−6 V).

Figure 3c shows the change in the ON/OFF current ratio of the device with the external voltage when a compliance current was applied in both directions. According to the figure, the ON/OFF current ratio of the Al/TH/ITO/PET device was greater than 10^2^ for a voltage difference of −1 V→1.25 V. The larger the ON/OFF current ratio is, the lower the misreading rate in circuit applications. Similarly, as shown in Figure 3d, the retention time of the device at a constant voltage of 0.50 V was tested. The retention time exceeded 10^4^ s, with the resistance state remaining stable, indicating that the device showed good data retention capabilities.

Then, the threshold voltage distribution was analyzed. Figure 3e shows histograms of the positive and negative threshold switching voltages (V_SET_), and Figure 3f is a statistical diagram of V_SET_. The threshold voltage distribution of the device was relatively centralized, indicating that it showed high stability for circuit applications.

**Figure 4:**
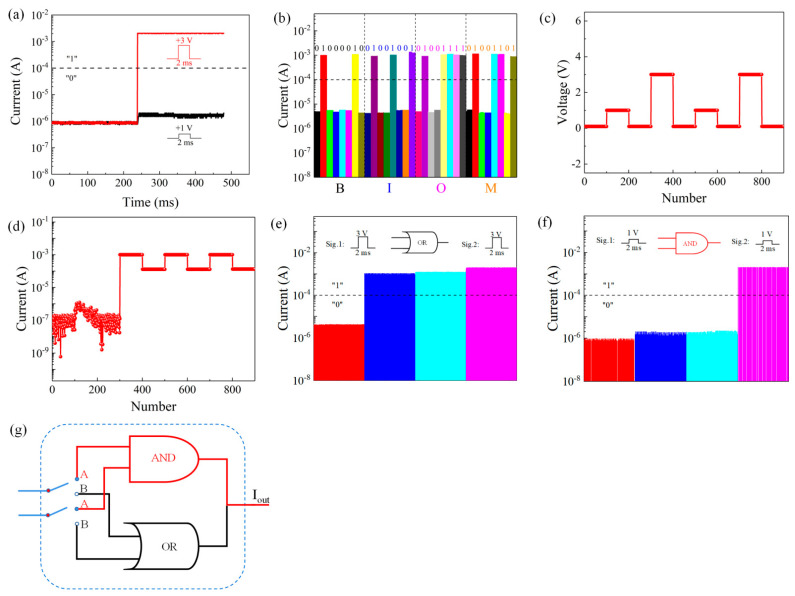
(**a**) Two input signals. (**b**) logic display of the word “BIOM”. Current response of the device under continuous pulse application: (**c**) voltage input and (**d**) current response. (**e**) “OR” gate. (**f**) “AND” gate. (**g**) working principle of the logic cell.

The resistance state transition of an Al/TH/ITO/PET device is closely related to the magnitude of the externally applied voltage. Thus, an external applied voltage can be used to obtain the output resistance state of a device. Figure 4a shows that when the applied pulse signal (+3 V) exceeded the threshold voltage, the current was greater than 10^−4^ A, and the corresponding current state was defined as a logical “1”. Otherwise, the current state was defined as a logical “0”. When a pulse signal of +1 V was applied, the current was approximately 1.66 × 10^−6^ A (logical “0”).

The current of the proposed device responds differently to different input signals and shows good retention characteristics. Therefore, it is possible to use the WORM characteristics of the device to implement a logical display function, as shown in Figure 4b. In the ACSII code table, the capital letter “B” is represented by “01000010”. Similarly, the letters “I”, “O”, and “M” correspond to different codes. Accordingly, the acronym “BIOM”, representing “biomemristor”, was chosen for a demonstration of the Al/TH/ITO/PET device cells.

The resistance state of a device depends on the magnitude of the externally applied signal. When the externally applied voltage exceeds the threshold voltage, the device changes from the HRS to the LRS. Thus, the response of the resistance state of a prepared device to various voltages was tested. With Icc = 1 mA, voltages of 0.3 V, 1 V, 0.3 V, 3 V, and 0.3 V were applied to the device, as shown in Figure 4c. Under the read voltage of 0.1 V, the initial state of the device did not change, i.e., it remained in the HRS. Then, when a 3 V voltage was applied to the device, the device switched to the LRS, as shown in Figure 4d.

Based on the different current responses of the device to different external applied signals, when an external pulse signal of +3 V is input separately or synergistically for 2 ms, the output current will exceed 10^−4^ A (logical “1”). Therefore, a logical “1” can be represented by one or two +3 V input signals. Otherwise, the logical state will be “0”. This output is equivalent to that of an “OR gate” in a logic gate circuit, as shown in Figure 4e, and the corresponding truth table is shown in Table 1.

An equally important gate circuit is an “AND gate”. As shown in Figure 4f, with an input signal of +1 V, only when the two input signals are +1 V simultaneously can the output current reach 10^−4^ A (logical “1”). Thus, only by applying two +1 V input signal to the device at the same time can the logical “1” state be realized. Otherwise, the logical state will be “0”. This output is equivalent to that of an “AND gate” in a logic gate circuit, and the corresponding truth table is shown in Table 2. Figure 4g shows a schematic diagram of the working principle of the logic cell. When one or two +3 V input signals are applied simultaneously to the device, the behavior of an “OR” logic gate can be realized. When two signals of +1 V are applied simultaneously, the behavior of an “AND” logic gate can be realized. Therefore, logic gates consisting of “OR gates” and “AND gates” can be implemented using flexible Al/TH/ITO/PET biomemristors.

**Figure 5:**
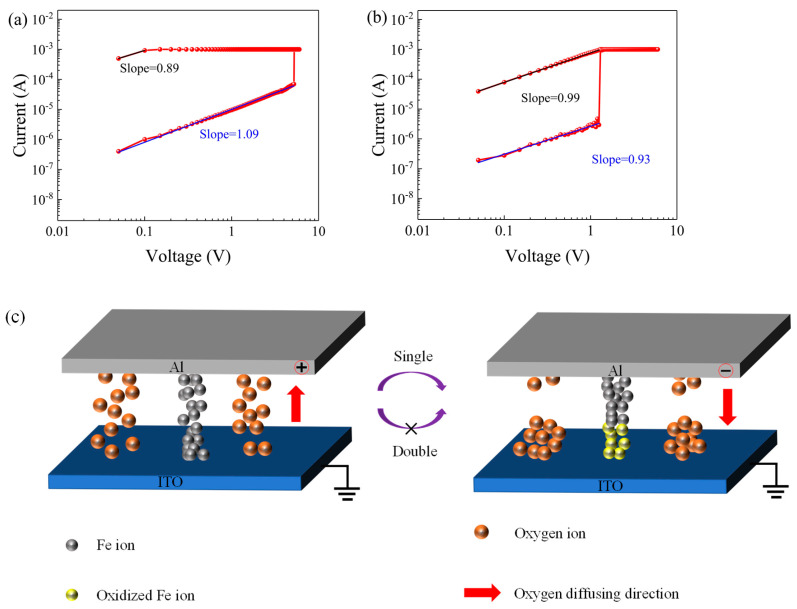
Log-log fits of the I–V curves at a positive bias: (**a**) compliance current in a positive scan. (**b**) compliance current in both a positive and negative scan. (**c**) schematic diagram of the resistance switching mechanism.

To explore the internal conductive mechanism of the device, the I-V characteristic curves measured under unidirectional and bidirectional compliance current scans were redrawn in log-log coordinates, as shown in Figure 5a,b. It can be seen from this figure that the fitted slopes of the HRS and LRS of the device were 0.89 and 1.09, respectively, under a compliance current in the positive scan direction. When the compliance current scan was applied in both directions, the fitted slopes of the HRS and LRS were 0.99 and 0.93, respectively. The fitted slopes in both cases were approximately 1, indicating that ohmic conduction was dominant. Therefore, it can be concluded that the conductive mechanism of the device is related to the formation and fracture of conductive filaments in the TH film.

The resistance state switching mechanism in this device can be attributed to the formation and fracture of conductive filaments, with the main cause being the migration of oxygen ions in the active layer and the oxidation-reduction reaction of metal cations. TH contains iron, calcium, copper [47] and other minerals. The work function of iron (4.50 eV) is slightly different from the work functions of both the top Al electrode (4.28 eV) and the bottom ITO electrode (4.70 eV). Therefore, iron ions can move between the top and bottom electrodes. When no compliance current is applied to the device in the negative direction and a positive voltage exceeding VSET is applied to the top electrode, due to the sufficiently large electric field, the negatively charged oxygen ions in the TH drift toward the top electrode, and the iron ions move toward the bottom electrode. The oxidized iron ions are reduced to form conductive filaments, and the device switches from the HRS to the LRS. In contrast, when a negative voltage exceeding V_RESET_ is applied to the top electrode, the opposite process occurs. The conductive filaments are disconnected, and the device recovers to the HRS and exhibits bipolar resistive switching characteristics. In the case in which a compliance current is applied in the negative direction, after the conductive filaments form, the externally applied energy is not sufficient to cause the conductive filaments to break. Thus, the device remains in the LRS and exhibits WORM characteristics.

## 4. Conclusions

In the study reported in this paper, Al/TH/ITO/PET sandwich-structured flexible RRAM devices were fabricated using a TH film as the active layer. When the compliance current was 1 mA in the positive voltage range and 100 mA in the negative voltage range, the proposed device showed bipolar resistive switching behavior. When a 1 mA compliance current was applied in a bidirectional voltage scan, the device displayed WORM characteristics, thereby enabling the realization of both “AND gates” and “OR gates” as storage logic gates. The device had a retention time of more than 10^4^ s and could work normally in a bent state. The device retained a good resistance switching ability after 10^4^ bending cycles. The resistance switching mechanism of the device is related to the formation and fracture of conductive filaments in the TH film. Thus, Al/TH/ITO/PET devices have high potential for use in flexible and green electronic devices.

## Figures and Tables

**Table 1:** OR gate.

Sig.1	Sig.2	Out
0	0	0
0	1	1
1	0	1
1	1	1

**Table 2:** AND gate.

Sig.1	Sig.2	Out
0	0	0
0	1	0
1	0	0
1	1	1
